# Association of αENaC p. Ala663Thr Gene Polymorphism With Sudden Sensorineural Hearing Loss

**DOI:** 10.3389/fgene.2021.659517

**Published:** 2021-12-22

**Authors:** Jialei Chen, Jing He, Jing Luo, Shixun Zhong

**Affiliations:** ^1^ Department of Otorhinolaryngology, The First Affiliated Hospital of Chongqing Medical University, Chongqing, China; ^2^ Department of Pathology and Pathophysiology, Chongqing Medical University, Chongqing, China

**Keywords:** sudden sensorineural hearing loss, ENaC, rs2228576, SNV, αENaC p.Ala663Thr

## Abstract

**Objective:** The etiology of sudden sensorineural hearing loss (SSNHL) is still unknown. It has been demonstrated that normal endolymph metabolism is essential for inner ear function and that epithelial sodium channels (ENaC) may play an important role in the regulation of endolymphatic Na^+^. This study aimed to explore the potential association between αENaC p. Ala663Thr gene polymorphism and SSNHL.

**Methods:** Polymerase chain reaction-restriction fragment length polymorphism (PCR-RFLP) was used to examine the genotype and allele frequency of the αENaC p. Ala663Thr polymorphism in 20 cases of low-frequency SSNHL (LF-SSNHL), 19 cases of high-frequency SSNHL (HF-SSNHL), 31 cases of all frequency SSNHL (AF-SSNHL), 42 cases of profound deafness SSNHL (PD-SSNHL), and 115 normal controls.

**Results:** The T663 allele was found to be significantly associated with an increased risk of LF-SSNHL (*p* = 0.046, OR = 2.16, 95% CI = 1.01–4.62). The TT genotype and T663 allele, on the other hand, conferred a protective effect for PD-SSNHL (AA vs. TT: *p* = 0.012, OR = 0.25, 95% CI = 0.08–0.74; A vs. T: *p* = 0.001, OR = 0.36, 95% CI = 0.21–0.61). However, there was no statistically significant difference in genotype or allele frequency between the two groups (HF-SSNHL and AF-SSNHL) and the control group.

**Conclusion:** The αENaC p. Ala663Thr gene polymorphism plays different roles in different types of SSNHL.

## Introduction

Sudden sensorineural hearing loss (SSNHL) is a common inner ear disease with unknown etiology. It is defined as a rapid and unexplained sensorineural hearing loss that occurs within 72 h and consists of a ≥20 dB reduction in hearing that affects at least two consecutive frequencies (Sun et al., 2018). Previous studies have reported that the incidence of SSNHL is 5–20/100,000 (Hughes et al., 1996), 275/100,000 (Teranishi et al., 2007), and 160–400/100,000 (Michel, 2011), in the United States, Japan, and Germany, respectively. The etiology and pathophysiological mechanism of SSNHL are still unknown. Nonetheless, it is hypothesized that SSNHL is caused by a combination of local and systemic factors. Vascular diseases (Mosnier et al., 2011), viral infections (Stokroos et al., 1998), autoimmune diseases (Berrocal and Ramírez-Camacho, 2002), and infectious diseases are the most common causes (Yoon et al., 2019).

The epithelial sodium channel (ENaC) is a complex composed of *α*, *β*, and *γ* subunits, with the *α* subunit (αENaC) playing a key role in channel function. ENaC is an important channel of Na^+^ because it allows Na^+^ to enter the cell passively. Previous research has shown that ENaC is a rate-limiting molecule that regulates Na^+^ transport (Kellenberger and Schild, 2002). ENaC is widely expressed in the inner ear, including the endolymphatic sac, stria vascularis, organ of Corti, and spiral ganglion, implying that ENaC is associated with endolymphatic metabolism (Zhong and Liu, 2004).

The epithelial sodium channel (ENaC) is a complex composed of *α*, *β*, and *γ* subunits, which are encoded by the *SCNN1A*, *SCNN1B,* and *SCNN1G* genes, respectively. Loss-of-function mutations in the *α* subunit of ENaC and deletion or mutation of PY group at -COOH terminal of *β* or *γ* subunit of ENaC can result in type 1 pseudoaldosteronism (PHA1) and Liddle syndrome, respectively (Hansson et al., 1995; Strautnieks et al., 1996).

The polymorphism of p. Thr663Ala (c.1987A > G) in *SCNN1A* is found naturally in humans. The substitution of alanine (A) for threonine (T) at the carboxyl terminus of the subunit occurs when adenine (A) in exon 13 of the *SCNN1A* gene is changed to guanine (G). Tong et al. (2006) reported that despite being initially reported as a threonine, the most common residue at 663 in humans is alanine with the polymorphism p. Ala663Thr. The activity of the ENaC with T663 (mutant type) was significantly higher than that of wild type (A663). The increase in activity was caused primarily by an increase in the number of active ENaC channels on the plasma membrane. Samaha et al. (2004) reported that p. Ala663Thr polymorphism increased the number of ENaC, and increased the current of ENaC significantly after the mutation. This polymorphism is thought to have a direct effect on the intracellular degradation and activity of sodium channels in human epithelial cells. Recent research has linked the p. Ala663Thr polymorphism to several physiological functions, including blood pressure (Yang et al., 2015), blood glucose metabolism (Guo et al., 2005), taste perception (Noh et al., 2013), and pulmonary function (Baker et al., 2011), among others.

SSNHL, as a multifactorial disease, may be linked to both environmental and genetic changes. A previous meta-analysis study found several disease susceptibility genes in SSNHL over the last 20 years, including inherited prothrombotic risk factors and their related genes, inflammation-related genes, tumor-related genes, oxidative stress-related genes, and hearing loss-related genes (Cao et al., 2019). To the best of our knowledge, only a few studies have investigated inner ear receptor genes. The purpose of this study was to investigate the role of ENaC gene polymorphism in SSNHL.

## Materials and Methods

### Study Subjects

A total of 112 patients with SSNHL referred to the Department of Otorhinolaryngology at the First Affiliated Hospital of Chongqing Medical University from September 2019 to November 2020 were enrolled. The diagnosis was made using the Chinese Medical Association’s 2015 guidelines. SSNHL patients were divided into four subgroups based on the frequency and degree of their hearing loss: 20 patients with low-frequency SSNHL (LF-SSNHL) with hearing loss below 1,000 Hz or at both 250 and 500 Hz hearing loss ≥20 dBHL; 19 patients with high-frequency SSNHL (HF-SSNHL) with hearing loss above 2000 Hz or at both 4,000 and 8,000 Hz hearing loss ≥20 dBHL; 31 patients with all-frequency SSNHL (AF-SSNHL) with hearing loss at all frequencies and an average hearing threshold between 250 and 8,000 Hz ≤ 80 dBHL; 42 patients with profound-deafness SSNHL (PD-SSNHN) with hearing loss at all frequencies and an average hearing threshold between 250 and 8,000 Hz ≥ 81 dBHL. The control group included 115 age-and gender-matched healthy volunteers from the same area who underwent a comprehensive physical examination and had no hearing loss during regular check-ups. Table 1 summarizes the clinical characteristics of SSNHL patients and healthy controls.

**TABLE 1:** Clinical features and demographic characteristics in SSNHL patients and healthy controls.

Parameter	LF-SSNHL group (*n* = 20)	HF-SSNHL group (*n* = 19)	AL-SSNHL group (*n* = 31)	PD-SSNHL group (*n* = 42)	Control group (*n* = 115)
Age (years)	36.4 ± 9.7	42.4 ± 18.4	47.2 ± 14.8	45.6 ± 14.4	43.3 ± 15.1
Sex (F/M)	8/12	7/12	14/17	23/19	55/60

Age is shown as means ± standard deviations. LF- SSNHL group = low frequency SSNHL group; HF- SSNHL group = high frequency SSNHL group; AF- SSNHL group = all frequency SSNHL group; PD- SSNHL group = profound deafness SSNHL group.

Patients were excluded from this study if they had a history of any of the following medical conditions: apoplexy, cancer, otitis media, Meniere disease, acute or chronic inflammation, virus infection, immunological disease, endocrine diseases, nervous system disease, infectious diseases, blood system disease, genetic disease, trauma, drug poisoning, diabetes mellitus, hypertension, pulmonary disease, taste aversion, or whose medical history was lacking. All SSNHL patients had unilateral onset and received treatment within 7 days of onset, and underwent a pure-tone hearing test during the first visit. Pure tone audiometry was used to determine bone and air conduction thresholds of 250, 500, 1000, 2,000, 4,000 and 8,000 Hz. In principle, asteroid (intravenous dexamethasone 10 mg/day tapered and maintained for at least 1 week), a fibrinogen-lowering drug (Batroxobin, 5U, qod, 10U for the first time, use when fibrinogen is greater than 1 g/L), and a neurotrophic factor (Extract of Ginkgo Biloba Leaves, 80 mg, tid, maintained for at least 1 month) were administered. Furthermore, the prognosis after 1 month of treatment was defined as follows: good prognosis (the hearing at the impaired frequencies is restored to normal, or has reached the level of healthy ears, or has returned to the level before suffering, or the average improvement of hearing at the impaired frequencies is more than 30 dB); and poor prognosis (the average improvement of hearing at the impaired frequency is less than 30 dB).

### Ethics Statement

The Ethics Committee of the First Affiliated Hospital of Chongqing Medical University (Chongqing, China), reviewed and approved the research, and all subjects provided written informed consent.

### Extraction of DNA From Blood

All blood samples were drawn from the antecubital vein. The genomic DNA was extracted from the blood clots using the Sangon Biotech Ezup Column Blood Genomic DNA Purification Kit (Sangon Biotech). The eluted DNA solution was then collected and stored at −20°C.

### Genotyping

The primers for the *SCNN1A* gene were designed using Primer Premier 5. The primer sequences were as follows: 1:5′ ACC​AGA​GTG​TTC​AGA​GGC​AGT​AC 3′, 2:5′ CCC​CAT​GTC​TCT​GTC​CTT​GTC 3′, and were synthesized by Sangon Biotech. The PCR amplification reaction conditions were as follows: a 5 min initial denaturation at 94°C, followed by 38 cycles each comprising of a 30 s denaturation at 94°C, 30 s annealing at 58°C, and 60 s extension at 72°C, and finally a 7 min extension at 72°C. The PCR products were digested directly for 3 h with restriction endonuclease ExoI, followed by 2.0% agarose gel electrophoresis. The genotypes of the products were analyzed using the gel image system.

### Verification of Genotyping by Sanger Sequencing

Sanger sequencing was performed on all participant DNA by Sangon Biotech Company (Shanghai, China) to validate the mutation detected in our experiment. PCR amplified fragments was subjected to Sanger Sequencing based on 3730XL DNA Analyzer (Applied Biosystems, United States). Sequence was analyzed using SeqMan (DNAStar, United States).

### Statistical Analysis

The SPSS20.0 statistical software package (SPSS Company, Chicago, Illinois, United States) was used to analyze all the data. A chi-square (χ^2^) test was used to compare categorical variables and to determine whether single nucleotide variants (SNV) genotype frequency was in Hardy- Weinberg equilibrium which was used to assess the quality of the genotyping data. The student’s t-test and analysis of variance (ANOVA) were used to compare continuous variables differences between two groups. In addition, the odds ratio was calculated using Binary Logistic regression analysis, and the relative risk of each genotype and allele was expressed by the 95% confidence interval. *p* < 0.05 was considered to be statistically significant.

## Results

### Clinical Characteristics

Table 1 displays the overall data for the SSNHL and control groups. There was no statistically significant difference in age (F = 1.871, *p* = 0.117) or gender (χ^2^ = 1.899, *p* = 0.687) between the SSNHL groups and the control group.

### p.Ala663Thr Gene Polymorphism’s Genotype and Allele Frequency Distributions

The χ^2^ goodness-of-fit test was used to examine the genotype frequency distribution of the p. Ala663Thr gene in the SSNHL and control groups. The findings revealed that both groups were in agreement with the Hardy-Weinberg equilibrium (*p* > 0.05). The genotype detected by Sanger sequencing were consistent with those detected by us. Table 2 displays the Binary Logistic regression results for SSNHL risk. The frequency of the T663 allele was significantly higher in the LF-SSNHL group than in the control group, and it was significantly associated with increased risk of LF-SSNHL (*p* = 0.046, OR = 2.16, 95% CI = 1.01–4.62). Furthermore, the TT genotype and T663 allele frequencies were significantly lower in the PD-SSNHL group than in the control group, indicating a protective effect for the PD-SSNHL (AA vs. TT: *p* = 0.012,OR = 0.25, 95% CI = 0.08–0.74; A vs. T:*p* = 0.001,OR = 0.36, 95% CI = 0.21–0.61). The p. Ala663Thr genotype and allele frequency did not differ significantly between the other two groups (HF-SSNHL group and AF-SSNHL group) and the control group (*p* > 0.05).

**TABLE 2:** Frequencies of alleles and genotypes of p.Ala663Thr polymorphisms in SSNHL patients and controls. The age and sex factors were adjusted in the multivariate logistic regression model.

Gene type	Control group (%)	LF-SSNHL group (%)	HF-SSNHL group (%)	AF-SSNHL group (%)	PD-SSNHL group (%)	P (vs. control)	Adjusted OR (95% CI)
AA	24 (20.8)	1 (5.0)	4 (21.1)	6 (19.4)	17 (40.5)	0.152^a^	1.00a (Ref.)^a^
						0.902^b^	1.00b (Ref.)^b^
						0.835^c^	1.00c (Ref.)^c^
						0.038*^d^	1.00d (Ref.)^d^
AT	57 (49.6)	9 (45.0)	10 (52.6)	16 (51.6)	19 (45.2)	0.262^a^	3.45 (0.39–30.03)^a^
						0.825^b^	1.15 (0.32–4.16)^b^
						0.570^c^	1.36 (0.46–4.04)^c^
						0.103^d^	0.49 (0.21–1.15)^d^
TT	34 (29.6)	10 (50.0)	5 (26.3)	9 (29.0)	6 (14.3)	0.083^a^	6.71 (0.77–57.95)^a^
						0.865^b^	0.88 (0.21–3.71)^b^
						0.824^c^	1.14 (0.35–3.71)^c^
						0.012*^d^	0.25 (0.08–0.74)^d^
A	105 (45.7)	11 (27.5)	18 (47.3)	28 (45.2)	53 (63.1)	0.046*^a^	0.46 (0.21–0.98)^a^
						0.831^b^	1.07 (0.53–2.17)^b^
						0.436^c^	1.16 (0.79–1.68)^c^
						0.001*^d^	2.77 (1.62–4.74)^d^
T	125 (54.3)	29 (72.5)	20 (52.7)	34(54.8)	31 (36.9)	0.046*^a^	2.16 (1.01–4.62)^a^
						0.831^b^	0.92 (0.46–1.86)^b^
						0.436^c^	0.86 (0.59–1.25)^c^
						0.001*^d^	0.36 (0.21–0.61)^d^

a Comparison between LF-SSNHL, patients and controls.

b Comparison between HF-SSNHL, patients and controls.

c Comparison between AF-SSNHL, patients and controls.

d Comparison between PD-SSNHL, patients and controls. Ref., AA, genotype (wild type) of each site was used as a reference. P and Adjusted OR (95% CI) values are all compared to the control group.

*p < 0.05.

### Factors Influencing LF-SSNHL and PD-SSNHL Prognosis

Age, gender, time from symptom onset to treatment initiation start, PTA before treatment, and allele type were considered as potential prognostic factors for inclusion in the binary logistic regression analysis. A total of 20 LF-SSNHL and 42 PD-SSNHL patients were treated according to the guidelines. A total of 14 had a good prognosis and 8 had a poor prognosis. There were 17 PD-SSNHL patients with a good prognosis and 25 with a poor prognosis. Table 3 shows the results: time from symptom onset to treatment initiation start was an independent risk factor for the prognosis of LF-SSNHL (*p* = 0.02,OR = 1.97, 95% CI = 1.11–2.43), while carrying the T allele was an independent protective factor for the prognosis of PD-SSNHL (*p* = 0.04,OR = 0.33, 95% CI = 0.12–0.95).

**TABLE 3:** Treatment outcome.

Parameter	LF-SSNHL group					PD-SSNHL group				
	B	S.E.	Wald	P	Adjusted OR (95%CI)	B	S.E.	Wald	P	Adjusted OR (95%CI)
PTA before treatment	0.10	0.07	2.08	0.14	1.11 (0.96–1.68)	0.02	0.02	1.29	0.25	1.01 (0.98–1.05)
Time from symptom onset to treatment initiation start	0.66	0.28	5.30	0.02*	1.97 (1.11–2.43)*	−0.27	0.05	0.29	0.58	0.97 (0.88–1.07)
Age	−0.27	0.07	0.14	0.70	0.97 (0.03–3.06)	−0.11	0.02	0.40	0.52	0.98 (0.95–1.02)
Gender										
Male					1.0(Ref.)					1.0(Ref.)
Female	−0.10	0.59	0.03	0.85	0.89 (0.28–2.86)	0.79	0.48	2.68	0.10	2.21 (0.85–5.75)
Allele distribution										
A					1.0(Ref.)					1.0(Ref.)
T	1.16	1.16	0.99	0.31	3.20 (0.32–31.42)	−1.10	0.57	4.23	0.04*	0.33 (0.12–0.95)*

*p < 0.05.

## Discussion

A large number of studies have been conducted to investigate the possible pathogenesis of SSNHL. Even though different treatment regimens for SSNHL have been reported, the prognosis is quite different, implying that the pathogenesis of different types of SSNHL may be different. Previous research has suggested that the majority of LF-SSNHL cases are caused by membranous labyrinth hydrops, the HF-SSNHL may be caused by hair cell injury, AF-SSNHL may be caused by stria vascular dysfunction or vasospasm of the inner ear, and the PD-SSNHL is caused by vascular embolism or thrombosis in the inner ear (Michel, 2011).

Endolymph is characterized by a high concentration of K^+^ (150–180 mM) and a low concentration of Na^+^ (<1 mM), resulting in the generation of endocochlear potential (+80 mv). The dynamic balance of the endolymph is primarily determined by the secretory and/or absorption activities of the inner ear’s special epithelial cells (Wangemann, 2002). This homeostasis must be strictly maintained to convert sound transduction into neural signals. The availability of K^+^ is critical in this transduction (Peters et al., 2004). K^+^ is taken up by the bottom marginal cells via Na^+^-K^+^-ATPase (a1b2 and a1b1) and Na^+^-K^+^-2Cl-(NKCC1) co-transporters, and secreted into the endolymph via apical K^+^ channels (KCNQ1/KCNE1 complex) (Peters et al., 2001). As a result, the Na^+^ excreted from the base of the marginal cells drives the Na^+^-K^+^-2Cl^-^ co-transporter, increasing the flow of K^+^ into the cells. When Na^+^ supply is disrupted, the absorption of K^+^ by Na^+^-K^+^-ATPase in the marginal cells is limited (Delpire et al., 1999). Endolymph Na^+^ absorption is primarily dependent on ENaC and the sodium hydrogen exchanger (NHE3) located in the marginal cells (Bond et al., 1998; Couloigner et al., 2001). Previous research has suggested that the p. Ala663Thr polymorphism may disrupt Na^+^ metabolism (Samaha et al., 2004; Tong et al., 2006). ENaC activity is high, supplying more Na^+^ to the endolymph. Hearing can be harmed if the endolymph volume or composition is abnormal.

Tran et al. (Tran Ba Huy, 2002) discovered the mRNA for the K^+^ channel ether à go-go (EAG) in rat cochleas and spiral ligament fibrocytes. They proposed that the EAG channels regulate K^+^ fluxes from the spiral ligament. Furthermore, they developed the concept of endolymphatic deafness and attempted to explain how SSNHL occurs through electrochemical changes in the endolymph. This is consistent with our previous research that found ENaC to be widely expressed in the spiral margin, spiral ligament, organ of Corti, and Reissner’s membrane, but not in the stria vascularis. Moreover, the expressions of Sgk1 and Af9, two key molecules involved in ENaC regulation, were shown in the aforementioned sites. Endolymphatic Na^+^ regulation is closely linked to the expression of ENaC and its regulatory molecules (Zhong et al., 2014).

Until now, the role of Na^+^ metabolism in SSNHL patients has remained unknown. Two questions can help understand the disorder of Na^+^ metabolism in SSNHL. Is a defect in Na^+^ metabolism the cause of SSNHL? Or is a disorder of Na^+^ metabolism a type of SSNHL pathological change?

This study found that the frequency of the T663 allele was much higher in the LF-SSNHL group than in the normal control group, implying that LF-SSNHL patients had higher ENaC activity. Currently, a large number of studies have shown the presence of endolymphatic hydrops (EH) in patients with LF-SSNHL, indicating that EH is closely associated with acute low-tone sensorineural hearing loss. We hypothesize that an increase in ENaC activity is one of the causes of EH, which may result in the occurrence of LF-SSNHL. However, the fact that the T allele was not associated with treatment outcome in LF-SSNHL patients suggested that EH may not be a prognostic factor in SSNHL. Both LF-SSNHL and Meniere’s disease have low-frequency hearing loss and EH, but their clinical symptoms, treatments, and therapeutic effects are very different (Chen and Young, 2006). EH can be caused by damage to endolymphatic secretory structures such as the stria vascularis, spiral ligament, and supporting cells. EH can also be caused by a systemic inflammatory, allergic, or autoimmune response (Zheng et al., 2019). As a result, we conclude that EH is, at least in part, the pathological change of LF-SSNHL, which may be induced by high ENaC activity. Similar findings have been reported by other authors (Cho et al., 2013; Ferster et al., 2017).

Another finding was that the frequency of the TT genotype and T663 allele was significantly lower in the PD-SSNHL group than in the control group, and the T allele was an independent protective factor for the prognosis of PD-SSNHL. Among the four SSNHL subtypes, PD-SSNHL has the most severe hearing loss and the poorest treatment effect. Impaired microcirculation in the inner ear caused by vasospasm or embolism can eventually lead to varying degrees of inflammation, and inflammation can cause microvascular damage and atherosclerosis, increasing the risk of ischemia. Because the blood supply to the cochlea is primarily dependent on a single vagus artery, ischemia and hypoxia can easily damage inner ear function, which may be one of the pathogenic mechanisms of PD-SSNHL. In conclusion, a lack of blood supply and low ENaC activity in the inner ear may reduce Na^+^ absorption, limiting K^+^ transport and thus causing PD-SSNHL, which is associated with more severe hearing loss and a poor prognosis.

This study has several potential limitations. Firstly, the sample size was small, and the collection of retrospective data was not under the control of the researchers who analyzed the data, resulting in unavoidable deviations. In addition, we did not look into other risk factors for SSNHL, such as vascular and environmental factors. Furthermore, studies in other populations, as well as evidence supported by basic science, are needed to determine the etiological contribution of aENaC p. Ala663Thr to SSNHL.

In conclusion, this study investigated the relationship between αENaC p. Ala663Thr gene polymorphism and SSNHL for the first time. In combination with previous research (Samaha, Tong), it is possible to conclude that αENaC p. Ala663Thr may protect patients of the PD-SSNHL while increasing the risk in patients of the LF-SSNHL. Our findings could shed more light on the pathogenesis of SSNHL.

## Data Availability

The raw data supporting the conclusion of this article will be made available by the authors, without undue reservation.
