# Corrigendum: Association of αENaC p. Ala663Thr Gene Polymorphism With Sudden Sensorineural Hearing Loss

**DOI:** 10.3389/fgene.2022.931037

**Published:** 2022-06-09

**Authors:** Jialei Chen, Jing He, Jing Luo, Shixun Zhong

**Affiliations:** ^1^ Department of Otorhinolaryngology, The First Affiliated Hospital of Chongqing Medical University, Chongqing, China; ^2^ Department of Pathology and Pathophysiology, Chongqing Medical University, Chongqing, China

**Keywords:** sudden sensorineural hearing loss, ENaC, rs2228576, SNV, αENaC p.Ala663Thr

In the published article, there was an error in affiliation for Jialei Chen. Instead of “^2^Department of Pathology and Pathophysiology, Chongqing Medical University, Chongqing, China,” it should be “^1^Department of Otorhinolaryngology, The First Affiliated Hospital of Chongqing Medical University, Chongqing, China.”

The authors apologize for this error and state that this does not change the scientific conclusions of the article in any way. The original article has been updated.

